# Influence of Benzoates of Alkalies on the Excretion of Uric Acid

**Published:** 1883-08

**Authors:** 


### Influence of Benzoates of Alkalies on the Excretion of Uric Acid.

Dr. Garrod, in his third Lumleian lecture, as reported in the *British Medical Journal*, April 21st, 1883, gives a summary of some interesting experiments on the influence of hippuric and benzoic acids on the elimination of uric acid, the conclusion he arrives at being that these two former, when in dilute solution, and alkaline, are capable of destroying the latter as a chemical experiment, and, when administered medically, of materially lessening the excretion. He gives some sets of experiments, which show that benzoates, when administered in set doses, have the effect of diminishing the uric acid. I have repeated his experiments so far as regards the administration of benzoates, and do not get results similar to his. On May 13th, the urine was collected, the diet having been regulated exactly for seven or eight days previously. The collections of 14th and succeeding days are as given in the following table:

| Date. | Urine. | Specific Gravity. | Uric Acid. |
|---|---|---|---|
| May 14th | 38 ozs. | 1020 | 13 grains. |
| " 15th | 36 " | 1022 | 12.3 " |
| " 16th | 26 " | 1030 | 13.0 " |
| " 17th | 29 " | Not analyzed.... | |
| " 18th | 20 " | 1030 | 13.2 " |
| " 19th | 32 " | 1023 | 13.0 " |
| " 20th | 38 " | Not analyzed.... | |
| " 21st | 40 " | 1023 | 12.4 " |
| " 22d | 48 " | 1020 | 13.7 " |
| " 23d | 40 " | 1026 | 14.0 " |
| " 24th | 32 " | 1025 | 13.2 " |
| " 25th | 28 " | 1025 | Not analyzed. |

On the 20th, 30 grains benzoate of soda in solution were taken at night in one dose. On the 21st, three doses, of 20 grains each, of benzoate of soda, 6 ounces extra water. On the 22d, two doses of 20 grains. On the 23d, three doses of 20 grains. On the 24th, none.

The benzoate acted somewhat as a diuretic, although very little extra water was taken. The estimations of uric acid given were made by the zinc sulphate precipitation process; and to corroborate, quantities of the urine were precipitated by the zinc sulphate solution after removal of the phosphates, and the precipitate, washed and decomposed by dilute sulphuric acid, gave abundant crystals of uric acid, as proved by microscope and murexide test.

Results so divergent as these are from those of Dr. Garrod, certainly surprised me; for although I have not seen his paper to the Royal Society, I have followed his directions closely, as given in his lecture-report. His process for estimation, as given in his first lecture, is the acidulation process, and I sought in this some explanation of our difference.

A portion of the urine collection of the 21st, and also of the 22d, 23d, and 24th, were set aside after acidulation. These urines gave very little crystallization of uric acid—a scarcely perceptible trace appearing even after seventy-two hours' standing, with occasional stirring. Of the urine collection of the 21st, 20 ounces were acidulated; and, on the 24th, the crystallization from the whole 20 ounces was scarcely ponderable, but some feathery crystals were evident, probably benzoic acid.

From this it is evident that the presence of a benzoate, or even of benzoic acid, can prevent the crystallization of uric acid from urine; but it is also evident that it does not destroy it; and this result is in agreement with those of Kerner, and of Wöhler and Keller, as quoted by Parkes.

But Dr. Garrod states in his lecture that the uric acid is destroyed, and states that the murexide test failed to answer, after a mixture of a benzoate and a urate in alkaline solution had stood some time at a temperature of the body. He states that a dilute solution is necessary, and from twenty-five to fifty times as much benzoate as urate. In his lecture, he gives nothing more definite than a dilute solution, but I took uric acid, 2 grains, benzoic acid, 60 grains, water, 4 ounces, and dissolved till clear with caustic soda, the solution being alkaline to test-paper; the whole was put into a four-ounce bottle, and kept for nine hours at a temperature of 98° Fahr. One-half of this mixture, diluted and precipitated by zinc sulphate solution, gave nearly one grain of uric acid. The other half, diluted again to 4 ounces, was kept for twelve hours at a temperature of 110° Fahr., and then gave very markedly the murexide test in a single evaporated drop. It was left twelve hours more at 100° Fahr., and again tested, no change being apparent. It was then rendered more alkaline with a mixture of potassic and sodic carbonate, and left twelve hours at 100°, at the end of which time, one drop, evaporated as before, gave faint evidence of uric acid by the murexide test.

Again, a weighed quantity of 1 grain of uric acid and 60 grains of benzoic acid were dissolved in 6 ounces of water with soda to a clear solution, and left 12 hours at a temperature of 100°. One drop gave clearly the murexide test. A little mixed sodic and potassic carbonates were added, and the mixture again left 12 hours at 100°.. There was now no evidence of uric acid, by the murexide test; but on precipitation with zinc sulphate solution, and solution of the precipitate in dilute sulphuric acid, abundant crystals deposited, of which a minute quantity gave very markedly the murexide test.

Again, a weighed quantity of one grain of uric acid was dissolved in 6 ounces of water by means of sodic and potassic carbonates in slight excess; and one drop of the solution evaporated rapidly (as in the other testings), gave a far fainter test spot of murexide than the drop from the same strength solution of the preceding experiment prior to digestion. This solution was then set aside, at 100° Fahr., for ten hours. One drop then evaporated gave very slight evidence of uric acid by murexide test. After twelve hours, no evidence of uric acid could be obtained by this test.

The results of the above experiments seem to point to the fact that the uric acid is not destroyed by benzoic acid, but exists in a state of combination (it may be as a compound acid), and when this combination is evaporated with nitric acid it gives no murexide with alkalies, and when it is precipitated by zinc sulphate solution, the uric acid combines with the zinc oxide. And the last experiment seems to demonstrate that an alkaline solution containing soda and potassa is capable of really destroying uric acid if kept at a temperature 98° Fahr., even without the presence of benzoic or hippuric acids, probably by oxydation. This was a discovery of Schunk, who found the products of decomposition to be oxaluric acid, and finally urea and oxalic acid; and Dr. Basham has noticed that this takes place in the animal economy when potash salts are administered, coincidently with the diminution of the uric acid (*Practitioner*, vol. v, 1870).

It is well known that there are many substances which, when present in a solution of uric acid, are capable of increasing its power of remaining dissolved, and benzoic acid certainly appears to be one of these.

I have not tried the effect of the administration of hippuric acid by mouth, because it is well known that if benzoic acid be administered, hippuric acid appears in the urine; and if this hippuric acid, formed in the animal, did not diminish the uric acid (though it did prevent its precipitation by acid), neither would hippuric acid administered by the mouth be likely to do so. Moreover, we have no guarantee whatever that hippuric acid, administered by the mouth, will appear unchanged by the urine; and, reasoning from the behavior of uric acid when so administered or injected, it would certainly not do so.

It is tolerably certain that variations in diet will cause variations in the excretion of uric acid; and, if a strong vegetable variation in the diet of mixed feeders will cause it to be replaced by hippuric acid, it is reasonable to suppose that the same cause, acting in a less degree, will produce the same effect in a less degree; therefore, we may conclude that, in the urine of mixed feeders, we have more or less of a substance which will prevent the precipitation of free uric acid; that the quantity, and therefore the effect, varies with the diet. It must be evident, therefore, that a process of estimation, capable of being affected in this manner, cannot be reliable.—*Edmund Alleyne Cook, in the British Medical Journal.*

